# RA-XTNet: A Novel CNN Model to Predict Rheumatoid Arthritis from Hand Radiographs and Thermal Images: A Comparison with CNN Transformer and Quantum Computing

**DOI:** 10.3390/diagnostics14171911

**Published:** 2024-08-30

**Authors:** Ahalya R. Kesavapillai, Shabnam M. Aslam, Snekhalatha Umapathy, Fadiyah Almutairi

**Affiliations:** 1 Department of Biomedical Engineering, SRM Institute of Science and Technology, College of Engineering and Technology, Chennai 603203, India; ahalya991@gmail.com; 2 Department of Biomedical Engineering, Easwari Engineering College, Ramapuram, Chennai 600089, India; 3 Department of Information Technology, College of Computer and Information Sciences (CCIS), Majmaah University, Al Majmaah 11952, Saudi Arabia; 4 Department of Information System, College of Computer and Information Sciences (CCIS), Majmaah University, Al Majmaah 11952, Saudi Arabia; fma.almutairi@mu.edu.sa

**Keywords:** UNet++, transformers, convolutional neural networks, rheumatoid arthritis

## Abstract

The aim and objective of the research are to develop an automated diagnosis system for the prediction of rheumatoid arthritis (RA) based on artificial intelligence (AI) and quantum computing for hand radiographs and thermal images. The hand radiographs and thermal images were segmented using a UNet++ model and color-based k-means clustering technique, respectively. The attributes from the segmented regions were generated using the Speeded-Up Robust Features (SURF) feature extractor and classification was performed using k-star and Hoeffding classifiers. For the ground truth and the predicted test image, the study utilizing UNet++ segmentation achieved a pixel-wise accuracy of 98.75%, an intersection over union (IoU) of 0.87, and a dice coefficient of 0.86, indicating a high level of similarity. The custom RA-X-ray thermal imaging (XTNet) surpassed all the models for the detection of RA with a classification accuracy of 90% and 93% for X-ray and thermal imaging modalities, respectively. Furthermore, the study employed quantum support vector machine (QSVM) as a quantum computing approach which yielded an accuracy of 93.75% and 87.5% for the detection of RA from hand X-ray and thermal images. In addition, vision transformer (ViT) was employed to classify RA which obtained an accuracy of 80% for hand X-rays and 90% for thermal images. Thus, depending on the performance measures, the RA-XTNet model can be used as an effective automated diagnostic method to diagnose RA accurately and rapidly in hand radiographs and thermal images.

## 1. Introduction

Rheumatoid arthritis (RA) is a chronic autoimmune disorder that mainly affects the musculoskeletal system over a prolonged duration [1]. If the disease is disregarded in the early stages, it has an impact on the patient’s physical function [2]. RA causes damage in the small joints, bone erosion, fatigue, and joint space narrowing. It presents significant challenges due to its chronic and debilitating nature, leading to joint inflammation, pain, and potentially severe physical disability if not managed early. The complexity of RA diagnosis and management is exacerbated by the variability in disease presentation and progression, as well as the limitations in existing diagnostic methods, particularly in detecting early-stage disease. Traditional diagnostic approaches, including clinical evaluation and imaging techniques, often fail to provide the necessary sensitivity and specificity for early detection.

Early diagnosis is crucial as it allows for timely intervention, potentially preventing irreversible joint damage and improving the patient’s quality of life. Common differential diagnoses include osteoarthritis and psoriatic arthritis, which share overlapping symptoms such as joint pain and swelling. However, these conditions differ in their underlying pathology and management strategies, making accurate diagnosis essential.

Radiography is considered as the conventional modality for diagnosing RA [3]. But using radiography, the premature changes due to RA are difficult to evaluate in small finger joints. Thermography, a non-invasive method, can be employed as a pre-screening system for the early identification of RA [4]. It provides an increased temperature profile in the regions affected by the synovial inflammation due to RA. Existing imaging techniques, including MRI and ultrasound, have been widely used in the evaluation of RA. MRI provides detailed images of soft tissues and is highly sensitive in detecting early joint changes, while ultrasound offers real-time assessment of synovitis and is cost-effective. Despite their advantages, these modalities have limitations such as high cost, limited accessibility, and operator dependency, which can affect diagnostic accuracy and consistency. This study, therefore, focuses on alternative imaging methods, specifically hand radiographs and thermal imaging, to address these challenges.

Thermography has been explored as a tool for detecting RA, particularly in its early stages. Among these findings, studies in orthopedics have shown that infrared thermography (IRT) is a sensitive and dependable technique for diagnosing, assessing, and monitoring osteoarthritis and rheumatoid conditions affecting various joints [5]. Advanced infrared thermal imaging cameras, known for their high temperature sensitivity, generate high-resolution thermal images [6]. These modern thermal cameras are highly advanced, compact, and portable, enabling quick image capture in short intervals, which provides an advantage over more labor-intensive imaging methods like ultrasonography [7].

Artificial intelligence (AI) has become a mainstream technique for medical image analysis, segmentation, and classification of various diseases [8]. Machine learning (ML), a subdiscipline of AI, handles a broad range of data, such as medical images, clinical data, demographic values, and temperature values from the thermograms to facilitate the automated detection of RA [9]. Deep learning (DL) is a subbranch of AI that uses deep layers, automated feature extraction, and classification [10]. Recently, the convolutional neural network (CNN) has become a well-known architecture for image processing.

Several researchers developed various techniques to detect RA based on ML and DL applications. Bai et al. predicted RA using features such as age, sex, and clinical data based on an artificial neural network (ANN) [11]. They employed a 5-fold cross-validation method to identify the model’s performance. The current approach used features from hand radiographs and thermal images rather than using demographic and clinical values. Also, a novel DL model was developed purely for the RA application. Morita and their co-researchers [12] used a support vector machine (SVM) to detect RA in hand X-ray images. They extracted the histogram of oriented gradients (HoG) features from the patches of the finger joints of X-ray images, and classification was carried out using an SVM classifier. They employed radiograph patches covering every joint in their investigation. However, the proximal interphalangeal (PIP) and metacarpophalangeal (MCP) are the finger joints that are most impacted. We used automatic segmentation with the UNet++ DL architecture in our investigation. Similarly, our work used automated feature extraction applying DL models in place of manually crafting feature extraction strategies. Ureten et al. built CNN architecture for the automated detection of RA from hand radiographs [13]. They employed You Only Look Once (YOLO) to detect the finger joints, and classification was performed using a pre-trained VGG-16 network. The weights from the ImageNet dataset are used in pre-trained models, which are ineffective for classifying RA. The current study used a novel DL architecture RA-XTNet that solely works for RA application. From the study by Wang et al., it is observed that the authors developed a computer-aided diagnosis (CAD) algorithm for the evaluation of RA from hand X-rays [14]. The authors proposed an automated labeling system by employing the YOLOv4 algorithm to predict RA. Their study only detected the finger joints, and classification was not performed. Our study classified the normal and RA subjects and developed a new architecture which was compared with the state-of-the-art models.

Mills et al. developed a diagnostic tool for the early prediction of RA using ML models [15]. The authors utilized clinical data as features for the classification using a machine learning model. They used clinical data instead of utilizing the crucial features from the images. Fung et al. developed a DL-based joint detection model in RA hand radiographs to identify the damaged joints [16]. The authors employed a YOLO object detection model to identify the PIP and MCP joints. The authors only detected the regions and further processing was not performed in their study which is one of the limitations of their study. Deimel et al. generated a DL model for the automatic scoring of X-ray progressions in RA patients [17]. They used a DL model for the joint extraction that takes labeling into account for both appearance and spatial connection. They obtained nominal values of specificity and sensitivity of 0.86 and 0.87 for the joint space narrowing in PIP joints which is one of their limitations. Izumi et al. developed an ensemble detection of hand joint ankylosis, subluxation, and dislocation using deep learning techniques [18]. The authors used a single shot multi-box detector (SSD) an object detection tool to identify the hand joints. After that, these images were fed to the ensemble predictor to detect ankyloses, subluxation, and dislocation of the hand joints. The limitation of their study is the quality of the dataset regarding the instability in annotation labels because of the features of the mTSS, which compares and assesses the condition before and after medication.

There is limited literature on using deep learning techniques on thermal images to detect RA. A study [19] demonstrated an ML model using the temperature measured from the small joints to predict RA. They fed the measured temperature values as an input to the ML models, including bagging, Adaboost, and random space, to classify RA and control groups. Most of the thermal imaging investigations employed temperature profiles from finger joints to identify RA [20,21]. The present approach extracted the high-level features from the thermal images and developed an architecture that solely works for RA application. The study by [22] developed an efficient DL-based system for the identification of RA in thermal images. They integrated a CNN and long short-term memory (LSTM) for the categorization of RA and controlled participants.

Transformers represent cutting-edge technology in computer vision, excelling in tasks such as image categorization, object detection, and semantic segmentation [23]. It is a deep learning architecture that adopts an encoder–decoder structure. Vision transformer (ViT) is the recently developed transformer that is used for image recognition [24]. Limited studies have employed ViT for the prediction of control and RA groups. Another state-of-the-art method with less computation time is quantum computing [25,26,27,28]. Quantum computers are square root times overhead compared to traditional computers. The integration of quantum computing and machine learning has set up a new era of research. A traditional ML kernel solves non-linear issues using a linear approach. On the contrary, quantum kernels analyze the kernel matrix for training and map the characteristics into a quantum-enhanced space.

The critical need for innovative approaches in RA diagnosis is underscored by the limited success of current methodologies. Existing machine learning (ML) and deep learning (DL) models have struggled with the accurate classification of RA due to the complexity of the disease and the challenges associated with distinguishing it from other forms of arthritis. Moreover, there is a scarcity of studies leveraging advanced technologies like quantum computing and transformer-based models for RA detection. In response to these challenges, this study proposes a novel approach utilizing a specialized CNN model, the RA-XTNet, which integrates quantum computing and transformer-based techniques. This innovative method aims to enhance the accuracy and efficiency of RA diagnosis, particularly from hand X-ray and thermal images. There are limited studies that compare the performance measures of radiographs and thermal images for the prediction of RA. The proposed work is innovative in that it uses the CNN transformer and the quantum computing approach to identify RA from thermal and hand X-ray images. The existing literature has not detected RA based on quantum computing and CNN transformer from hand radiographs and thermograms. Demographic and clinical values were used in most of the ML classification studies to identify RA. The accuracy attained was quite low even though customized models were employed in the literature that is currently available. In addition, a small amount of data and many epochs were used to train the existing models. Moreover, no custom model has been employed in any literature to classify RA from thermal images. The present method employed crucial features extracted from hand X-ray and thermal images, rather than using demographic and clinical data. Also, by employing data augmentation techniques, the current work trained the models with a larger dataset and fewer epochs. Transformer and quantum-computing-based techniques for the classification of RA are fewer. The proposed study developed a specialized CNN model designed for predicting RA using hand X-rays and thermal images. Our aim and goal of the research are (i) to examine and contrast the performance metrics of the radiographs and thermal images based on AI and quantum computing techniques for the prediction of RA; (ii) to build a custom CNN model for the categorization of RA; (iii) to compare the performance measures of ML, DL, transformer, and quantum computing models in the context of RA detection. The novelty of the proposed research lies in the following aspects:

- An automated segmentation technique, UNet++, was employed to separate the PIP and MCP joints.
- A dedicated CNN architecture named RA-XTNet was designed for the prediction of RA from hand X-ray and thermal images.
- In addition, a state-of-the-art method, ViT, was implemented that identifies RA in hand radiographs and thermal images.
- Finally, a quantum model called QSVM was utilized for the classification of RA based on hand X-rays and thermograms.

The organization of the current study is as follows: the first section describes RA, AI, quantum computing, and several literature reviews based on the detection of RA from radiographs and thermograms. The second section demonstrates the various methodologies such as UNet++, color-based k-means, ML, DL, CNN transformer, and quantum computing techniques. The performance measures of the various models based on radiographs and thermal images are explained in the third section. The fourth section gives the discussion and conclusion of the present approach.

## 2. Materials and Methods

### 2.1. Volunteers

The study had one hundred individuals, fifty of them were RA patients and the other fifty were healthy participants. The current study acquired 200 radiographs from both the hands of the control and RA participants. Similarly, from the same subjects 200 thermal images were acquired. The rheumatologist’s recommendations were followed while choosing the RA patients for the planned investigation. To identify RA in the one hundred participants, thermal and real-time hand radiographs were acquired. Informed permission was obtained from all participants, and the planned work was granted approval from the Institute Ethics Committee (Human Studies) of SRM Medical College, Hospital and Research Centre, Kattankulathur, Tamil Nadu, India (Approval Number: 2449/IEC/2021). The volunteers were classified into two groups: healthy individuals (mean age ± standard deviation: 46.4 ± 12.4 years) and those with RA (mean age ± standard deviation: 45.4 ± 13.4 years). RA was confirmed by an expert rheumatologist of the Department of Rheumatology, SRM Hospital and Research Centre based on the Indian Rheumatology Association criteria [29]. Individuals with osteoarthritis, psoriatic arthritis, recent physical therapy, fractures, diabetes mellitus, hypertension, fever, or heart disease were excluded from the research. Hand X-ray reports that indicated findings suggestive of RA by the expert radiologist were available during the study.

### 2.2. Image Acquisition and Dataset

Real-time hand radiographs were obtained by an expert X-ray technician using a Siemens Pleophos D, 300 mA X-ray machine. The X-ray tube voltage was set to 60 kV with an exposure dose of 3 ms and film focus distance of 100 cm to capture both hands’ anterior–posterior (AP) view. The obtained radiographs measured 2328 × 2928 pixels and were saved in jpg format. Two hundred radiographs were obtained from both hands of healthy and RA patients to detect RA. From the same subjects, thermal images were acquired in a temperature-controlled (20 degree Celsius) dark room. The patients were asked to remove their ornaments and expose both hands in AP view during the image acquisition process. The current study employed a FLIR A305SC thermal camera for capturing thermal images. The researcher maintained a one-meter distance between the camera and the subject’s hand to acquire 200 images for normal and RA subjects.

Then, 200 images were divided into 160 images for training and 40 images for testing the DL models using an 80–20% split. To address the substantial data requirements of DL models, the study implemented five methods of data augmentation, resulting in an increase in training images to 800. The data augmentation techniques, such as rotation of 90 degrees, coarse dropout, scaling, transposition, and translation, were performed to increase the training images. The data augmentation techniques were employed in the 160 training images while the 40 test images were preserved as such. Thus, a total of 560, 240, and 40 images were utilized for training, validation, and testing phases in the DL and ViT classification. Radiographs and thermal images used the same data splitting mentioned above to classify RA.

**Figure 1:**
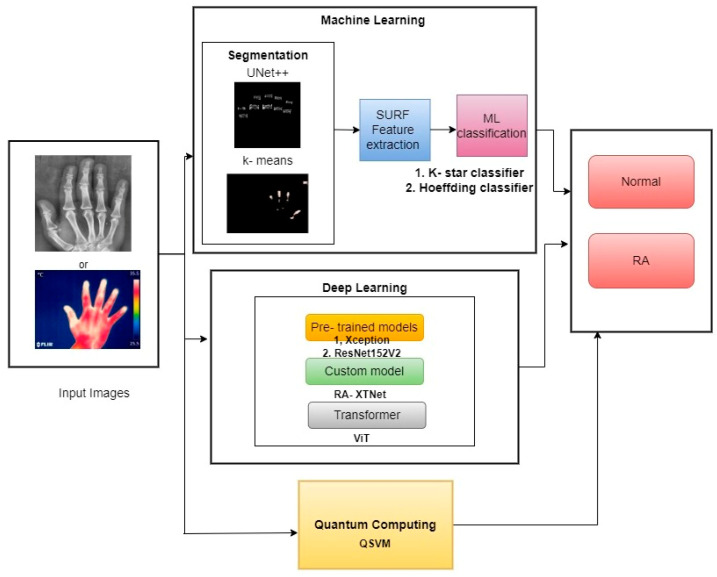
Block diagram of the proposed work in RA prediction and classification.

The schematic diagram of the current study is demonstrated in Figure 1. The study segmented the PIP and MCP finger joints from hand radiographs using UNet++ architecture, and the hot spot regions from thermal images were segmented using color-based k-means technique. The ML classifiers such as k-star and Hoeffding classifiers were used in the study for RA classification. Similarly, pre-trained classifiers such as Xception and ResNet152V2 models were utilized in the current study. A novel proposed model named RA-XTNet architecture was implemented in the study for the RA classification. Furthermore, ViT and QSVM models were used in the present approach to identify RA and healthy subjects.

### 2.3. Image Segmentation and Feature Extraction from Hand X-rays and Thermograms

For the hand radiographs, the annotations were performed by the rheumatologists. The ground truth images were generated using Matlab R2021b (Mathworks, Natick, MA, USA). The original image and ground truth images were fed to the UNet++ model for the segmentation of PIP and MCP joints. The study employed a deep-learning-based segmentation by UNet++ to segment the PIP and MCP finger joints. Initially, Fadiyah Almutairi images are pre-processed to enhance the clarity of bone structures and joints. This might involve techniques such as contrast enhancement, noise reduction, and normalization of pixel intensity values. The input image is passed through a series of convolutional layers with ReLU activation and max-pooling operations. This part of the network captures the contextual features by progressively reducing the spatial dimensions and increasing the depth of the feature maps. The features are then upsampled using transposed convolutions. The decoder path reconstructs the image from the encoded features, aiming to produce a segmented output with the same resolution as the input image. UNET++ differentiates itself by having nested skip connections that densely connect the encoder and decoder subnetworks. These connections help in bridging the semantic gap between the high-level and low-level features, improving the accuracy of the segmentation. The model is trained on a dataset of hand radiographs with annotated segmentation masks. The segmentation masks typically outline the bones and joints, which are the regions of interest. A combination of loss functions, such as Dice coefficient loss and cross-entropy loss, is used to optimize the network. These losses are crucial in ensuring the segmented output closely matches the ground truth. As the network learns, it becomes adept at identifying and segmenting the relevant anatomical structures in the hand radiographs, including the bone contours, joint spaces, and potential erosion. The final output is a segmented image where the bones and joints are clearly delineated. The segmentation highlights areas of interest for further analysis, such as the detection of RA-related changes.

UNet++ architecture is a modification of the UNet model used for biomedical image segmentation [30]. To improve segmentation accuracy, convolutional layers and dense blocks were added to the UNet++ model in between the encoder and decoder. In contrast to the conventional UNet architecture, UNet++ makes use of three add-ons: deep supervision, dense skip connections, and redesigned skip paths. UNet++’s deep supervision modifies model complexity, while balancing speed and performance. There are skip channels between the encoder and decoder layers in UNet++ thanks to its extensive skip connections. Ensuring that all previous attributes are gathered and arrive at the current node via the dense convolution block along each skip pathway is the main goal of these dense skip connections. This produces attributes with complete resolution at numerous semantic levels. The reduced semantic gap between the encoder and decoder layer properties is a result of the modified skip paths. UNet++ combines the preceding convolution layer’s output of the same dense block with the equivalent upsampled output of the lower dense block. When obtaining semantically similar attributes, the encoded feature’s semantic level becomes closer to that of the attributes in the decoder, facilitating the optimization process. One hundred normal and hundred mask images were used in the study for the UNet++ image segmentation. Similarly, the same number of images was employed to segment the finger joints in RA patients.

Thermal image segmentation was carried out using a color-based k-means clustering technique, as outlined in our previous work [31]. For the hand thermal images, segmentation of the hot spot was performed using the color-based k-means technique in Matlab R2021b. The optimal ‘k’ value of the current study was obtained using the Elbow method. A hand thermogram is captured, usually showing varying temperatures across different regions of the hand. These temperature variations are represented by different colors in the thermographic image. The thermogram, initially in RGB color space, is often converted into a different color space like hue, saturation, value (HSV) or lightness, A/B channels (LAB). This conversion helps in better distinguishing the temperature-based color variations. The image may be normalized to ensure consistent intensity levels across different thermograms, facilitating more effective clustering. The number of clusters (k = 5) is selected. For thermograms, this number typically corresponds to the different temperature ranges (e.g., cold, warm, hot, mild, background regions). Each pixel in the thermogram is treated as a data point with color features. The k-means algorithm groups these pixels into k clusters based on the similarity of their color features. The algorithm minimizes the variance within each cluster while maximizing the variance between clusters. The algorithm iteratively adjusts the cluster centers (centroids) and reassigns pixels to the nearest cluster until convergence is achieved (i.e., when the cluster assignments no longer change significantly). The result of the k-means clustering is a segmented image where pixels are grouped into distinct clusters, each corresponding to a specific temperature range. Each cluster is often assigned a different color to visually distinguish the regions with varying temperature levels on the hand thermogram. The segmented thermogram is analyzed to identify regions of abnormal temperature, which may indicate inflammation or other pathological conditions associated with rheumatoid arthritis (RA).

**Figure 2:**
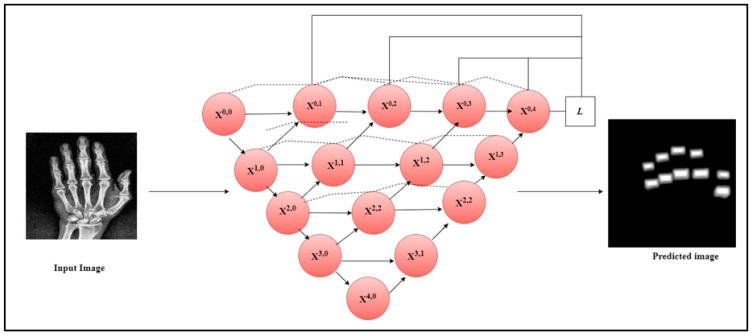
UNet++ architecture for the segmentation of PIP and MCP joints.

The current work utilized the Speeded-Up Robust Features (SURF) feature extractor to extract the crucial features from the segmented finger joints in radiographs and the hot spot regions from thermal images. SURF is a feature extractor that uses a Gaussian second derivative mask on an input image at different scales to exact the features [32]. It applies the mask across each axis at 45 degrees which makes the extractor fast and robust to rotation. Figure 2 illustrates the architecture diagram of the UNet++ model to segment the PIP and MCP joints from the hand radiographs. The original hand radiographs and the mask image were passed through the UNet++ architecture to segment the PIP and MCP joints.

### 2.4. Machine Learning Classification

The current study utilized labeled data, leading to the application of supervised learning techniques such as the Hoeffding tree and k-star algorithm for the classification of individuals with RA from those who are healthy. The Hoeffding tree classifier employs Hoeffding bounds to classify and analyze the decision trees [33]. Hoeffding bounds specify the number of instances needed to achieve a specific confidence level. For ‘k’ independent observations of a random variable ‘r’ with a range ‘R’, the Hoeffding bound ensures that, with confidence 1-∂, the true mean of ‘r’ is within r|-ε, where r| is the observed mean of the data samples and

ε = √(R^2^ ln(1⁄∂))/2k (1)

The current research utilized a Hoeffding threshold of 0.05, a batch size of 100, and a minimum fraction of weight information gain of 0.01. The leaf prediction strategy for the proposed Hoeffding tree used was the naïve Bayes adaptive method for the classification of RA. k-Star is an instance ML classifier which uses the entropy measure to determine the similarity function between a test and training instances [34]. The entropy measure is based on the likelihood of changing one instance into another by randomly choosing between all transformations. The study used k-star with a batch size of 100 and a global blend of 20 to identify control and RA patients.

### 2.5. Deep Learning Classification

To classify RA, the study employed modified pre-trained architectures like Xception and ResNet152V2 architectures. The Xception model, developed by Google, is known as the extreme version of the inception model [35]. It uses a modified depthwise separable convolution layer for classification purposes. The thermal and hand radiograph inputs were scaled to 256 × 256 pixels and passed to the multiple layers of the ResNet152V2 and Xception model to classify RA. ResNet152V2 is a pre-trained CNN model with 152 layers [36]. It is a modification of the ResNet design that solves the vanishing gradient problem by using skip connections. Skip connections are a feature of the ResNet architecture that allow the ReLU activation function of a layer to be connected directly to succeeding layers, without the intermediate layers. Numerous residual units are placed on top of it, and they are shown as

A_q_ = {A_q_, n|1 ≤ n ≤ N} (2)

Bq = h (xq) + f (xq, aq) (3)

xq + 1 = f (yp)(4)

where ‘Aq’ depicts the weight and bias of the qth residual block, xq input and xq + 1 is the output of the qth block, ‘N’ is the number of residual blocks, ‘F’ is the residual function, ‘f’ is the ReLU activation function, h(xq) = xq demonstrates the identity mapping. The modification to V2 involves creating a pathway within a residual block and throughout the entire model to facilitate feature propagation. Feature propagation occurs throughout the entire model for identity mappings h(xq) and f(yp). The equation for deeper block ‘Q’ and trivial block ‘q’ is provided as follows:

C_Q_ = x_q_ + ∑_(i = 1)^(Q − 1)F (x_i_, a_i_) (5)

where ‘CQ’ is the summation of x_0_ and precedes the residual function output.

The current study incorporated a global average pooling layer and two dense layers with 64 and 2 neurons into the pre-trained ResNet152V2 and Xception architecture.

**Figure 3:**
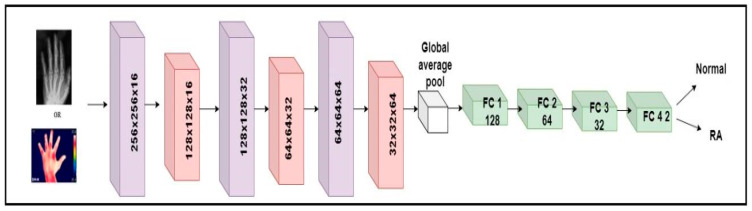
Architecture diagram of custom RA-XTNet for the categorization of RA.

**Figure 4:**
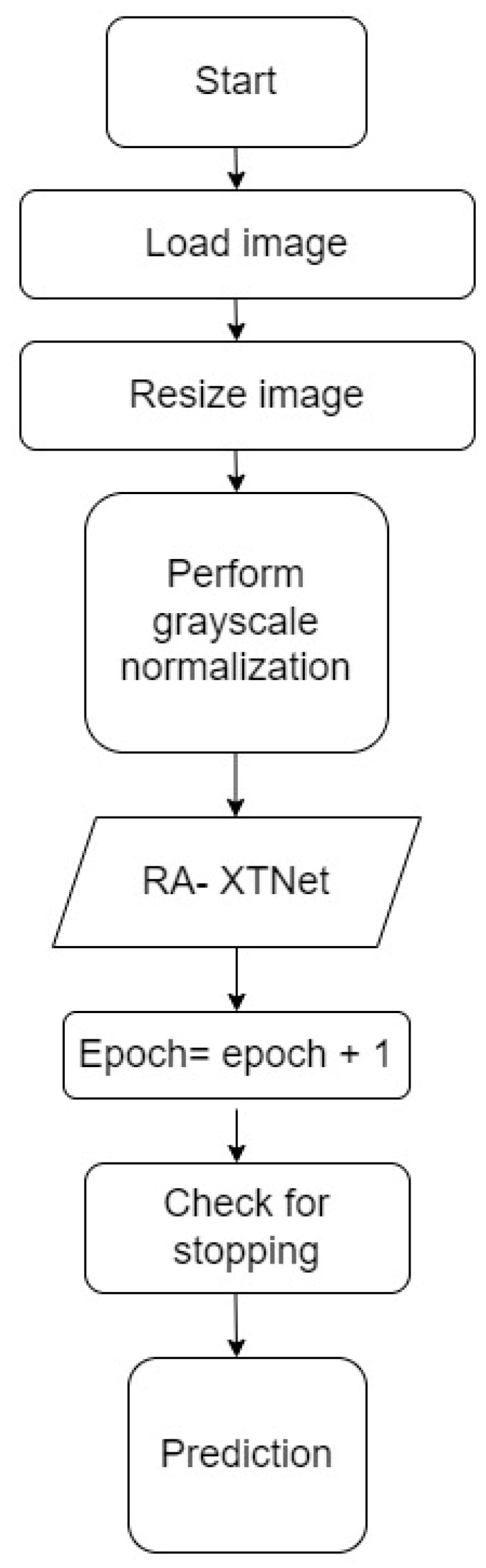
Flowchart of the RA-XTNet for RA prediction in hand radiograph and thermal images.

The millions of natural data available in the ImageNet database weights were used to learn the pre-trained CNN systems [37]. Despite changing the pre-trained models, it is unable to deliver consistent performance for RA application based on accuracy. To detect RA, a custom model named RA-XTNet was developed. The RA-XTNet model consists of three convolution layers, three max pool layers, a global averaging pool layer, and four fully connected (FC) layers. The input hand radiographs were scaled to 256 × 256 pixels and passed through the convolution layer with 16 neurons, a 3 × 3 mask area, and stride 1. Each convolution layer was followed by the ReLU and max pool layers with mask size 2 × 2 and stride 2. Since max pool is employed, the image is downsampled to 128 × 128 with 16 neurons. These attributes (features) were fed to the next convolution layer with 32 nodes and a mask size of 3 × 3. Again, these attributes were downsampled to 64 × 64 using a max pool layer of stride 2. After that, these attributes were passed to a convolution layer with 64 nodes and mask area of 3 × 3. The attributes from the convolution layer are fed to a max pool layer which downsamples the image to 32 × 32 pixels. The downsampled feature maps from the final max-pooling layers were input into a global average pooling, followed by four FC layers with 128, 64, 32, and 2 nodes, respectively. Similarly, the input infrared images were fed through the different layers of the RA-XTNet for the evaluation of RA. Figure 3 illustrates the architecture of RA-XTNet for the categorization of RA. Figure 4 represents the flowchart of the proposed RA-XTNet model for RA prediction. The detailed step by step procedure for RA-XTNet model is elaborated in Algorithm 1.
**Algorithm 1:** RA-XTNet modelStep 1: Load the images, train dataset = 800 images, test dataset = 40 images Step 2: Image resized to 256 × 256 pixels Step 3: Grayscale normalization of the input images Step 4: Train, validation split of 70–30% for the images in the train dataset Step 5: Build the RA-XTNet model Step 6: Initialize epoch = 1 Step 7: Train and validate the RA-XTNet model Step 8: Epoch = epoch + 1 Step 9: Check for stopping criteria                        if epoch ≤ 50                              repeat step 7 & 8                       else                             stop Step 10: Prediction                          for i = 1 to 40                                 predict the test image using RA-XTNet                                   compare the predicted labels with the actual labels                           stop

### 2.6. Transformer Classification

CNNs are excellent at extracting features but cannot efficiently encode the positional relationships of various features. The deepest levels of a CNN can only visualize what the upper tiers have passed down to them. Thus, overall context of the feature information is lost. Although adding more filters increases representation capacity, it increases the computational expenses. Researchers proposed several architectural modifications resulting in attention processes and providing an effective solution over time. The areas of the image that CNN should focus on are selected through the attention procedure and then forwarded to the more profound levels. Researchers use the accomplishment of the transformer network in processing natural language to power their computer vision studies. A transformer adaptation called ViT was proposed by [38] and may be used directly to extract fine-grained information from sequences of image patches. ViT resolves the long-range interdependence among visual material by applying global attention to 16 × 16 patches of the whole image, concentrating on the prominent global characteristics. It maximizes the attention approach without sacrificing the computing performance to include global context in the visual characteristics.

Transformers are widely used in NLP because they effectively transfer learning to downstream processes. The transformer architecture comprises an encoder and decoder block for image categorization. The encoder portion of the transformer is the sole component needed for image identification. To provide data to the encoder component of the transformer design, positional encoding is applied to an image patch embedding to maintain the patch order, and the resulting positional encoded patch insertions are then supplied to the encoder block. The encoder block comprises feed-forward layers, multi-head self-attention, layer normalization, and another layer of normalization followed by the feed-forward layer. Using query, key, and value matrices in the self-attention layer, attention values are calculated to assess the relationship between each patch. Several self-attention ratings are determined to provide a better representation, acting as a multi-head. The results of these heads are merged into a single vector, into which an input vector is included via a skip link, and normalization is applied. The result of this normalization is then transmitted to the feed-forward layer, where normalization is applied, and the output from the previous layer is added once more with the aid of skip connections. These skip links make the interaction between the representations of various levels possible. Several encoder blocks can be piled to gather in the image transformer. For classification purposes, the transformer encoder block’s output is directed to a classifier at the end.

**Figure 5:**
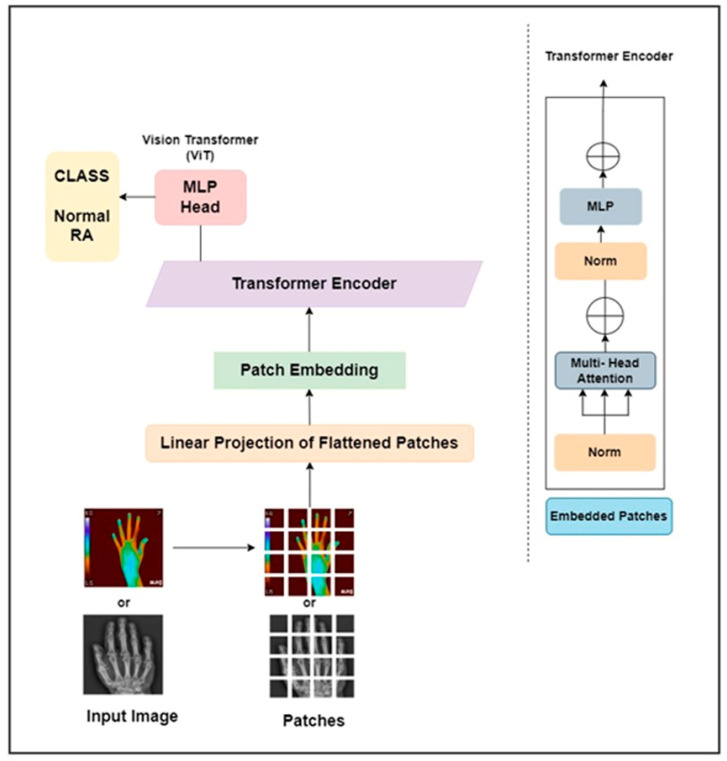
ViT architecture for the categorization of RA.

This work proposed a ViT as pure transformer architecture to detect RA. It is trained on the ImageNet and ImageNet 21k datasets. The input hand radiographs were scaled to 256 × 256 pixels and fed to the ViT model, which split the images into square patches of size 64. To create a vector representation of each image patch, the square patch was flattened and combined across the channels of the image. After that, a lower-dimensional linear embedding was developed from the flattened patches. The ViT model learns the pattern of the input images by adding a positional embedding to each patch. These patch embeddings were passed to piled transformer layers, and the output was fed to a multi-layer perceptron (MLP) to identify RA and non-RA subjects. Similarly, the infrared hand images were resized to 256 × 256 pixels and fed through the different layers of the ViT architecture to identify RA. The architectural diagram of the ViT approach to assess RA is elaborated in Figure 5. The hand radiographs and thermal images were passed through the ViT model for classification purposes.

### 2.7. Quantum Machine Learning Classification

The study used QSVM for the identification of RA and control groups [39]. In quantum computing, the classical data point x| is converted into quantum data point |ϕ (x|)›. This is achieved by using a quantum circuit U ϕ (x|) |0› where ϕ(x) is the classical function belonging to a classical data x|. The equation for the quantum circuit is given as:

(6) $$U_{\phi \;(x|)}=\exp\;(i\;\sum_{S\subseteq [n]}\phi_{s}\;(x|)\;\prod_{i\varepsilon S}Z_{i})$$

where Zi is the Pauli Z basics. In QSVM, a feature map is required to convert classical data into quantum data. The present study employed ZZFeature map for converting classical into quantum data.

### 2.8. Training and Validation

The current research utilized a Hoeffding threshold of 0.05, a batch size of 100, and a minimum fraction of weight information gain of 0.01. The leaf prediction strategy for the proposed Hoeffding tree used was the naïve Bayes adaptive method for the classification of RA. Also, for the k-star classifier, a batch size of 100 and a global blend of 20 was used to identify control and RA patients. One hundred normal radiographs and one hundred mask images were used in the study for the UNet++ image segmentation. Similarly, the same number of images was employed to segment the finger joints in RA patients. Among the 200 subjects, an 80–20% split was performed in which 160 images were used for training and 40 images for testing the UNet++ model. The model was trained using 500 epochs, batch size of 16, and learning rate of 0.001 (Table 1). The Adam optimizer and binary cross-entropy loss were used to train the UNet++ model for the segmentation of PIP and MCP joints. Similarly, the same number of images was used for the color-based k-means segmentation in thermal images. The ML classifier used a 10-fold cross-validation technique to classify RA and normal subjects. It was performed for 40 test segmented radiographs and thermal images.

The QSVM employed in the study used the same number of subjects as in ML classification for training (80%) and testing (20%) the model. From the total of 80 subjects, 64 subjects were employed for training and 16 subjects for testing. The same split was used for radiographs and thermal images to classify normal and RA patients using QSVM. The QSVM model was trained with a learning rate of 0.005, perturbation of 0.1, and simultaneous perturbation stochastic approximation (SPSA) optimizer (Table 2). DL and ViT classification were performed by using 200 images (normal and RA). Here, 200 images were divided into 160 images for training and 40 images for testing the DL models using an 80–20% split. To address the substantial data requirements of DL models, the study implemented five methods of data augmentation, resulting in an increase in training images to 800. Data augmentation techniques, such as rotation of 90 degrees, coarse dropout, scaling, transposition, and translation were performed to increase the training images. The data augmentation techniques were employed in the 160 training images while the 40 test images were preserved as such. Thus, a total of 560, 240, and 40 images were utilized for training, validation, and testing phases in the DL and ViT classification. Radiographs and thermal images used the same data splitting mentioned above to classify RA. The current study trained the pre-trained and custom models using a learning rate of 0.001, batch size of 16, and 10 epochs for pre-trained models and 50 for the custom net. The models were trained using a stochastic gradient descent (SGD) optimizer and binary cross-entropy loss function. The ViT architecture was trained using an AdamW optimizer, learning rate of 0.001, weight decay of 0.0001, batch size of 128, and 100 epochs (Table 3). The patch size of the ViT architecture was 64 × 64, patches per image were 16 and elements per patches were 12288. The last MLP consisted of two dense layers with 64, 32, and 2 neurons. The dataset was sampled using Monte Carlo cross-validation for the training and validity split using a test size of 0.3 and random state of 42.

## 3. Results

The baseline characteristics of the study were indicated in Table 4. The table shows that the RA group exhibits significantly higher levels of inflammatory markers (anti-citrullinated protein antibodies, ESR, CRP) which are the characteristic symptoms of RA compared to the normal group.

The entire process was carried out with Dell Vostro 14 3000 Intel Core i5 8th Gen, 8 GB RAM for the proposed study. The annotations and the generation of masks of the PIP and MCP joints were performed using the Matlab R2021b platform. Also, the color-based k-means clustering and SURF feature extraction were performed using Matlab R2021b. The UNet++ segmentation, DL, ViT, and QSVM were carried out in Google Colaboratory using Python programming. Libraries such as TensorFlow+ Keras were used for the pre-trained RA-XTNet model and UNet++ architecture. The ML classification was carried out in Weka software.

**Figure 6:**
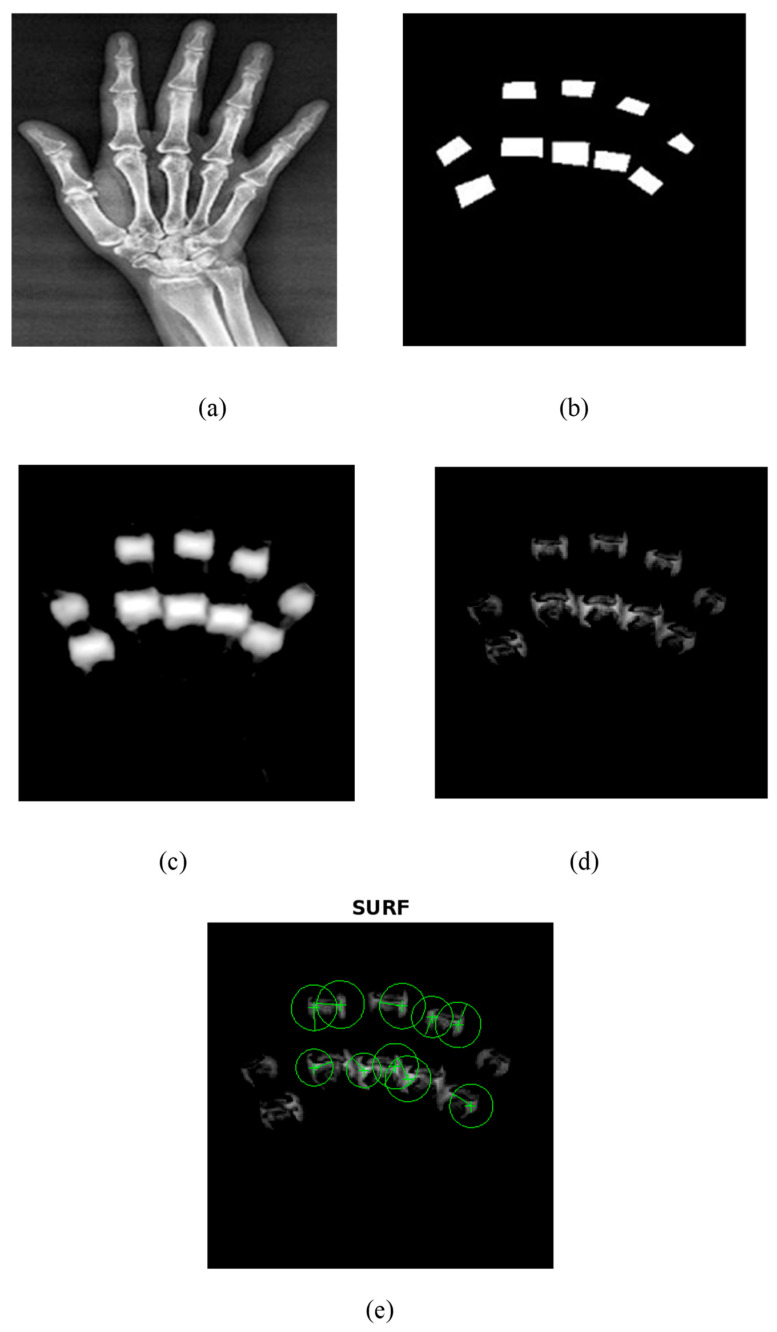
UNet++ segmentation and SURF feature extraction. (**a**) Input hand radiograph. (**b**) Ground truth image. (**c**) Predicted test image. (**d**) Subtracted image. (**e**) SURF feature extraction.

The proposed UNet++ segmentation method correctly segmented the PIP and MCP joints as shown in Figure 6. A train–test split of 80–20% was utilized in the current research to train the UNet++ model. The model was trained using 500 epochs, batch size of 16, and learning rate of 0.001. The Adam optimizer and binary cross-entropy loss were used to train the UNet++ model for the segmentation of PIP and MCP joints. Figure 6 depicts the UNet++ finger joint segmentation and SURF feature extraction. Figure 6a represents the hand radiograph, Figure 6b the ground truth image, Figure 6c the predicted image, Figure 6d the subtracted image, and Figure 6e the SURF features extracted from the grayscale subtracted image. The joints alone were segmented using UNet++ architecture as depicted in Figure 6d and SURF features were extracted for further classification. The present study employed an automated segmentation technique and only the features from the PIP and MCP joints from normal subjects and RA are extracted, which accurately classifies using the ML models.

As an initial step, PIP and MCP joints were annotated by an expert rheumatologist. Then from the annotated images, the ground truth images were generated by means of mask generation. The mask generation was carried out by using Apeer software (available online) for all the images. Then the original radiograph and the ground truth images were passed to the UNet++ architecture to segment PIP and MCP joints. The grayscale image of the segmented joints was obtained by subtracting the segmented binary image from the original image which is depicted in Figure 6.

The performance metrics of the UNet++ segmentation method are depicted in Table 5. For the ground truth and the predicted test image, the study yielded a pixelwise accuracy of 98.75%, intersection over union (IoU) of 0.87, and Dice coefficient of 0.86 that indicates a good similarity index. From the evaluation metrics, it was observed that the UNet++ segmentation results are in alignment with the ground truth image, which yields a good Dice coefficient and IOU.

**Figure 7:**
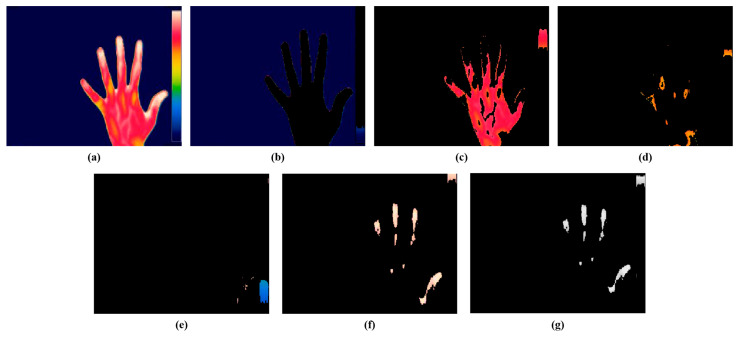
Color-based k-means segmentation. (**a**) Thermal hand RA image. (**b**) Cluster 1. (**c**) Cluster 2. (**d**) Cluster 3. (**e**) Cluster 4. (**f**) Cluster 5. (**g**) Gray output of cluster 5.

The present study employed a color-based k-means technique for the segmentation of the hot spot regions from thermal images. The optimal value of k for the study was 5, consistent with our prior research. Figure 7 represents the segmentation of hot spots from an RA hand thermal image. Figure 7b represents cluster 1 which shows the background of the input image, Figure 7c represents cluster 2 which segments the red color region having mild inflammation, Figure 7d indicates cluster 3 which segments the yellow region, Figure 7e depicts cluster 4 which segments the blue part, and Figure 7f shows cluster 5 which segments the white color representing the hot spot region in the RA thermal image. The hot spot will be absent for a normal hand thermal image. Figure 7e represents the gray output of cluster 5 for extracting the features. From the hot spot regions, SURF features were extracted for classification purposes. For further classification, the current study retrieved the characteristics from the hot spot regions induced by RA inflammation.

**Figure 8:**
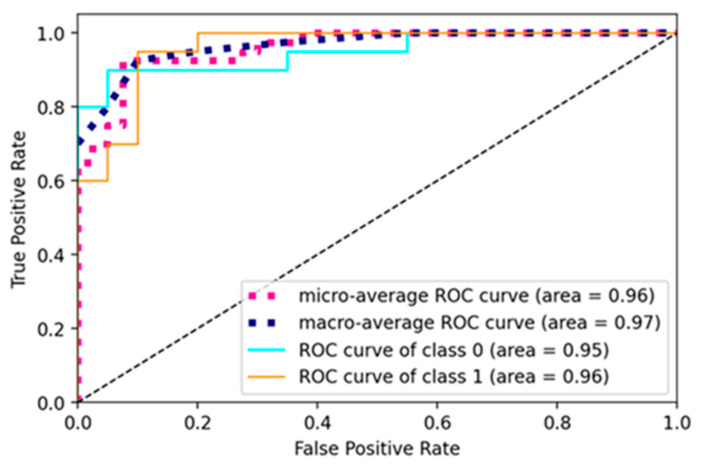
ROC curve of the RA-XTNet model of the thermal images.

The current study trained the pre-trained and custom model using a learning rate of 0.001, batch size of 16, and 10 epochs for pre-trained models and 50 for a custom net. The models were trained using the SGD optimizer and binary cross-entropy loss function. The ROC curve of the RA-XTNet to evaluate RA from thermal images is represented in Figure 8. The ROC value for class 0 (normal) was obtained as 0.95 and class 1 (RA) as 0.96 for the RA-XTNet architecture from the thermal images.

In the current study, the transformer model was trained with a learning rate of 0.001, weight decay of 0.0001, a batch size of 256, and a total of 100 epochs. The patch size of the input images was set to 64, and the number of transformers was 8.

**Figure 9:**
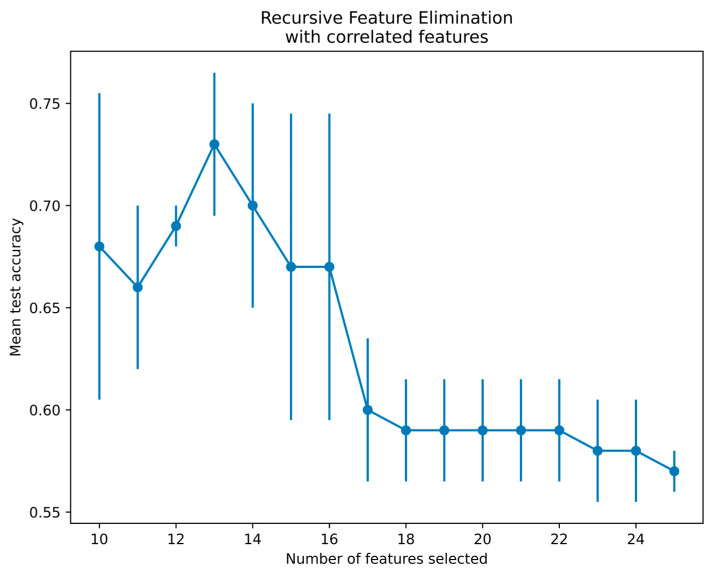
Feature selection using RFECV method for thermal images.

Since there is a limitation of using qubits in the qiskit platform, 25 features from the SURF extractor were reduced to 13 features using a recursive feature elimination with cross-validation (RFECV) technique for radiographs and thermal images [40]. The RFECV method eliminates the weakest features and selects the best subset of features for classification. The present study employed an SVM with linear kernel and stratified cross-validation with three folds to select the best features by the RFECV technique. Figure 9 demonstrates the thermal feature selection using the RFECV method for the QSVM classification. The features selected via RFECV for thermal images are depicted in Figure 9. Notably, at feature 13, there was a notable peak in mean test accuracy, followed by a subsequent decrease in accuracy. Thus, the optimal number of features selected was 13 and passed to the QSVM model for classification.

**Figure 10:**
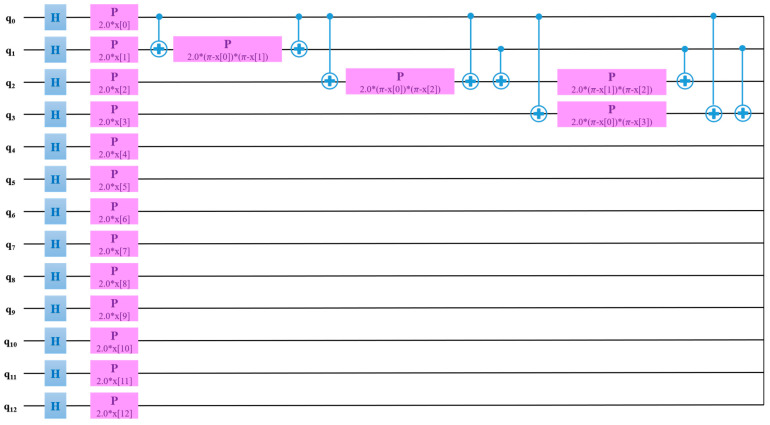
QSVM circuit for the classification of RA using thermal images.

The QSVM model was trained with a learning of 0.005, perturbation of 0.1, and simultaneous perturbation stochastic approximation (SPSA) optimizer. Thermal images outperformed radiographs for the classification of RA using the QSVM model. Figure 10 exhibits the QSVM circuit of thermal images for the classification of RA and healthy groups. In the proposed study, given the use of 13 features, a quantum circuit employed 13 qubits for classification purposes.

The performance analysis of the pre-trained models for the classification of RA is depicted in Table 6. From Table 6, it is evident that the models yielded low accuracy since no fine-tuning was performed for the detection of RA.

To fine-tune the models, four fully connected layers were added, because the actual pre-trained models do not perform adequately. Table 7 illustrates the comparison of pre- trained fine-tuned DL models, custom model, transformer, and quantum computing methods. For the classification of RA and healthy groups, the Hoeffding classifier outperformed the k-star classifier. It is evident from Table 4 that for thermal images, the classification accuracy was higher compared to radiographs. Also, from Table 7, custom the RA-XTNet model achieved the highest accuracy compared to the state-of-the-art models employed in the present approach. The features present in the thermal images are more prominent compared to those in the radiographs. Thus, the classification using thermal images outperformed radiographs in terms of performance accuracy.

Table 8 demonstrates the different performance characteristics of various models for the detection of RA. The Cohen’s kappa score is a statistical value that is used to calculate the interrater reliability of the classes. TPR/sensitivity represents the actual positive that is positive in the test, and FPR falsely rejects the true value. From Table 8, the sensitivity (total positive rate) of RA-XTNet architecture of thermal image is the highest compared to the state-of-the-art models. Also, the time complexity for the ViT was higher than that of other models.

This study demonstrates a comprehensive approach to diagnosing rheumatoid arthritis (RA) by leveraging AI and quantum computing techniques across two imaging modalities: radiographs and thermal images. The proximal interphalangeal (PIP) and metacarpophalangeal (MCP) joints were segmented using the UNet++ architecture, which is a variant of the U-Net model tailored for more accurate segmentation in medical imaging. Hot spot regions, indicative of inflammation, were segmented using a color-based k-means clustering technique. After segmentation, machine learning (ML) classification was performed by extracting features using the Speeded-Up Robust Features (SURF) extractor. Similarly, in thermal images, ML classification followed feature extraction. A unique pre-trained model, RA-XTNet, was employed for automatic feature extraction and classification of RA. This model achieved 90% accuracy in predicting RA from radiographs and 93% accuracy from thermal images, with corresponding sensitivities of 0.85 and 0.90. The QSVM model reached a classification accuracy of 87.5% for hand X-rays and 93.75% for thermograms. Vision-transformer-based architecture yielded classification accuracies of 80% for hand radiographs and 90% for thermal images. The study concludes that thermal imaging outperformed radiography in detecting RA, making it a valuable supportive modality. The RA-XTNet model is highlighted as a robust computer-aided diagnostic tool that could significantly aid clinicians in the early identification and diagnosis of RA.

## 4. Discussion

This work compared the performance of two modalities, hand radiographs and thermal images, based on AI and quantum computing techniques. The proposed work segmented the PIP and MCP joints from the hand radiographs using the UNet++ model. For the thermal images, the hot spot region was segmented by employing a k-means clustering technique. The features from both the segmented regions were extracted using the SURF feature extractor and ML classification utilizing k-star and Hoeffiding classifiers. The ML classification techniques require segmentation and manual feature extraction which is a tedious process. Also, from Table 5, it is evident that the performance of ML classification for the detection of RA is comparatively poor. Hence, in the proposed study, we employed pre-trained models such as Xception and ResNet152V2 for the automated feature extraction and classification. These models were trained on the massive ImageNet dataset and are less effective for RA diagnosis. Therefore, we developed a customized CNN RA-XTNet model to classify RA from hand radiographs and thermal images. The RA-XTNet model is built with fewer layers and the computational time and cost are less compared to the pre-trained models.

From Table 6, it is evident that the DL models trained from scratch have surpassed the ML models for the detection of RA from hand radiographs and thermal images. Even though the DL models outperformed the ML models, the attained accuracies were less. Therefore, fine-tuning of the DL models was performed by incorporating four fully connected layers with 128, 64, 32, and 2 neurons, respectively. From Table 7, the fine-tuned models yielded better accuracies compared to the models trained from scratch. Still, the accuracies attained were low, therefore, a custom RA-XTNet model was developed solely for the classification of RA.

The RA-XTNet model surpasses the ML and pre-trained models, while analyzing the crucial performance parameters. The RA-XTNet model yielded an accuracy of 90% and 92.5% for radiographs and thermal images, respectively, for the classification of RA. The study compared the performance of radiographs and thermal images based on ViT architecture. The ViT architecture based on thermal images attained high accuracy compared to the radiographs. Furthermore, comparisons of radiographs and thermal images were performed based on quantum computing. As depicted in Table 7, the QSVM model for thermal images outperformed the radiographs based on accuracy. The performance metrics comparison of the various models is shown in Table 8. From Table 8, the RA-XTNet model outperformed in terms of various metrics such as Cohen’s kappa, TPR, and FPR compared to the other models.

Summarizations of the existing literature are depicted in Table 9 for the detection of RA in hand radiographs and thermal images. Most of the studies based on ML classification employed demographic and clinical values to detect RA. The custom CNN models utilized in the literature based on hand radiographs yielded less accuracy compared to the proposed RA-XTNet model. As far as our knowledge extends, there is no literature that has utilized a custom CNN model for the automated classification of RA in thermal images.

Bonnin et al. employed AI techniques to identify RA in hand X-rays [41]. The authors automated the Sharp/van der Heijde (SvH) score in hand radiographs using CNN techniques. They divided the dataset into training, validation, and testing segments (64%, 16%, and 20%, respectively) to train the CNN system. To predict RA, the authors’ classification efficiency was 84%. Folle et al. used CT scans to diagnose RA, psoriatic arthritis, and control groups based on joints. The Grad CAM method was used by the authors to create heat maps of the important joints [42]. For classification, they suggested using a convolutional supervised autoencoder system. Using an autoencoder-based CNN system, the authors classified 86% of the patients as having RA, 11% as having psoriatic arthritis, and 3% as being in the control group.

RheumaViT was created in a study [43] to automatically assess the finger joints and forecast RA. Their model included a transformer to assess the bone degradation and JSN brought on by RA, as well as a regression phase for joint localization. Efficient Net for joint localization and TinyViT for joint evaluation were included in RheumaViT. By calculating the coordinates of each joint’s location in the hand radiographs, the joint localization procedure was carried out. To complete this assignment, bounding boxes that provide the coordinates of two opposed corners were created. An examination using TinyViT was used to obtain the erosion and JSN ratings.

Most of the research to identify RA in infrared scans using ML classification included clinical and demographic values. For the proposed model, an accuracy of 90% and 92.5% was obtained for hand radiographs and thermal images, respectively. The ViT architecture attained an accuracy of 80% and 90% for X-ray and thermal images to identify RA. To the best of our knowledge, no literature has used a customized CNN system to evaluate RA automatically in thermal pictures. As a result, comparing the results of radiographs and thermal imaging can be used to forecast healthy controls and RA as a pre-screening tool or auxiliary modality. Additionally, the suggested RA-XTNet and QSVM models may serve as a useful computerized diagnostic technique for RA estimation. The current study is constrained by the small amount of real-time data. To expand the data, the study also used traditional techniques for data augmentation. In the future, further data will be added, and a DL-based technique for data augmentation will be used to categorize RA.

In recent years, various studies have employed machine learning techniques to aid in the diagnosis of rheumatoid arthritis (RA). The authors of the current study propose that their approach, the RA-XTNet model, may be superior due to its integration of advanced technologies like transformers and quantum computing, which are designed to handle complex data and provide more accurate predictions.

The RA-XTNet model has shown promising results, particularly in differentiating RA from other conditions using hand radiographs and thermal images. However, direct comparisons with other studies are challenging due to differences in study design, datasets, and the stages of RA evaluated. For instance, while some studies focus on early RA diagnosis, others may include a broader range of disease stages, including more advanced cases. This variability makes it difficult to draw definitive conclusions about the relative effectiveness of different ML methods.

The stage of RA at the time of diagnosis is critical. Early-stage RA can be challenging to diagnose due to subtle symptoms and the lack of clear clinical signs, making it a prime target for advanced diagnostic methods. However, diagnosing RA in its later stages, typically after a few years of disease progression, is generally easier due to more pronounced symptoms and visible joint damage. As a result, the true test of any diagnostic tool or ML model lies in its ability to identify early-stage RA accurately.

The authors emphasize the need for their RA-XTNet model to be evaluated specifically for early-stage RA, as this is where the most significant clinical impact can be made. Early and accurate diagnosis allows for timely treatment interventions, which can prevent joint damage and improve long-term outcomes for patients. Therefore, while the RA-XTNet model appears promising, further validation against early-stage RA data is necessary to confirm its clinical utility and superiority over existing methods.

While the RA-XTNet model demonstrates good results in distinguishing between established RA cases and healthy controls, it is crucial to contextualize these findings and acknowledge the limitations inherent in the study design. The current study primarily involves a cross-sectional analysis of patients with established RA and a matched healthy control group. This design allows for the validation of the model’s ability to identify clear cases of RA versus non-RA individuals based on hand radiographs and thermal images. However, the study’s scope does not extend to predicting early RA, particularly among individuals in an ‘at-risk’ group with early or possible RA symptoms. The lack of longitudinal data and a defined ‘at-risk’ cohort limits the model’s application as a predictive tool for early diagnosis. Another limitation is that the tender joint counts and swollen joint counts were not formally recorded.

For a more robust validation, future research should involve the following: a well-characterized group of individuals with early, possible RA symptoms should be included. This group should be age- and sex-matched with a control group without joint pain or deformity. Detailed follow-up data would enable an assessment of how initial ML predictions align with clinical diagnoses over time. Tracking the progression of individuals from early symptoms to a confirmed diagnosis of RA (or other conditions) can provide critical insights into the model’s predictive accuracy. This approach would better assess the model’s ability to detect subtle early changes indicative of RA before they manifest into clinically obvious signs. The effectiveness of ML models heavily depends on the quality and representativeness of the training data. Including diverse patient profiles and ensuring high-quality imaging can improve the generalizability and reliability of the model. In future, a subsequent study might include formal documentation of tender and swollen joint counts to enhance the robustness of the findings. Future studies should ensure that all relevant clinical data, including joint counts, are systematically recorded and reported. Future studies could expand on the current findings by incorporating more comprehensive clinical assessments, including accurate tender and swollen joint counts, to provide a clearer picture of disease severity.

Thus, by comparing the performances of radiographs and thermal images, it was observed that thermal images can be employed as a pre-screening tool or supporting modality for the prediction of healthy and RA subjects. Furthermore, the proposed RA-XTNet and QSVM models can be used as an effective automated diagnostic tool for the detection of RA. The proposed RA-XTNet model, leveraging deep learning and quantum computing techniques, offers a novel approach by integrating these imaging modalities for more accessible and cost-effective RA detection.

## 5. Conclusions

The current study juxtaposed two imaging modalities, radiographs and thermal images, using AI and quantum computing methods. The PIP and MCP joints were segmented using UNet++ architecture for deep learning segmentation for the ML classification in radiographs. The hot spot regions in the thermal images were segmented using a color-based k-means clustering technique. ML classification was performed after extracting features using the SURF feature extractor. Additionally, the pre-trained and unique RA-XTNet model was used to perform automatic feature extraction and classification for the prediction of RA. The RA-XTNet model achieved 90% and 93% accuracy in predicting RA in radiographs and thermal imaging, respectively. It yielded a sensitivity of 0.85 and 0.90 for hand radiographs and thermal images. Quantum computing and transformer technology were also used in the study to classify RA. The QSVM model attained a classification accuracy of 87.5% and 93.75% for hand X-rays and thermograms. The ViT architecture yielded an accuracy of 80% and 90% for hand radiographs and thermal images to distinguish RA from healthy subjects. When compared to radiography, the thermal imaging performed better at detecting RA. To detect RA, thermal imaging can be used as a supportive modality. Additionally, the RA-XTNet model is a powerful computer-aided tool for the identification of RA which can assist clinicians in diagnosis. One critical limitation is the lack of evaluation of early-stage RA patients. The model’s effectiveness in detecting subtle signs of early inflammatory arthritis remains untested, which is a crucial aspect for early intervention and treatment. The model requires further validation, particularly through studies that include a more diverse patient population and follow-up for early arthritis cases. This is essential to determine its real-world applicability and robustness across different stages of RA.

## Figures and Tables

**Table 1:** Hyperparameters for UNet++ in hand radiograph segmentation.

Model	Hyperparameters					
UNet++	Random state	Learning rate	Batch size	Epochs	Optimizer	Loss
	42	0.001	16	100	Adam	Binary cross-entropy

**Table 2:** Hyperparameters for quantum support vector machine.

Model	Hyperparameters					
QSVM	Feature Dimension	Maximum iterations	Learning rate	Perturbation	Optimizer	Loss
	50	100	0.005	0.1	SPSA	SVC

**Table 3:** Hyperparameters for vision transformer.

Model	Hyperparameters						
ViT	Learning rate	Weight decay	Batch size	Transformer layers	Epochs	Optimizer	Loss
	0.001	0.0001	128	8	100	AdamW	Binary cross-entropy

**Table 4:** Demographic variables for normal subjects and RA patients.

Baseline Characteristics	Normal	RA
Age (years)	46.4 ± 2.4	45.4 ± 2.8
Duration of disease (years)	-	5.6 ± 1.5
Anti-citrullinated protein antibodies (U/mL)	35.5 ± 8.5	58.91 ± 10.8
Erythrocyte sedimentation rate (mm/hr)	23 ± 4.5	44.53 ± 6.5
C-reactive protein (mg/L)	10 ± 1.8	41.11 ± 2.6

**Table 5:** Evaluation metrics of UNet++ segmentation.

Model	IoU	MoU	Dice Co-Efficient	Pixel-Wise Accuracy
UNet++	0.87	0.87	0.86	98.57%

**Table 6:** Comparison of machine learning and pre-trained deep learning models trained without modifications in the layers.

Models	Radiographs					Thermal Images				
	Accuracy (%)	Precision	F1 Score	Recall	AUC	Accuracy (%)	Precision	F1 Score	Recall	AUC
*k*-star	58.2	0.58	0.58	0.58	0.60	62.5	0.63	0.61	0.62	0.63
Hoeffding	71.2	0.72	0.70	0.71	0.77	75	0.75	0.75	0.75	0.78
AlexNet	60	0.78	0.54	0.61	0.70	71	0.70	0.71	0.69	0.75
Xception	61.5	0.79	0.56	0.62	0.78	72.5	0.71	0.72	0.70	0.69
ResNet152V2	74.2	0.74	0.72	0.71	0.80	77	0.79	0.77	0.77	0.82

**Table 7:** Performance metrics analysis of the pre-trained models after fine-tuning, custom model, transformer, and quantum computing methods.

Models	Radiographs					Thermal Images				
	Accuracy (%)	Precision	F1 Score	Recall	AUC	Accuracy (%)	Precision	F1 Score	Recall	AUC
AlexNet	77	0.78	0.77	0.79	0.82	87	0.89	0.87	0.87	0.93
Xception	77.5	0.79	0.77	0.78	0.83	87.5	0.88	0.87	0.88	0.94
ResNet152V2	87.5	0.88	0.87	0.88	0.94	90	0.90	0.90	0.90	0.94
RA-XTNet	90	0.90	0.90	0.90	0.95	92.5	0.93	0.92	0.93	0.96
ViT	80	0.80	0.80	0.80	0.86	90	0.90	0.90	0.90	0.95
QSVM	87.5	1.0	0.85	0.75	0.8	93.75	0.88	0.93	1.0	0.90

**Table 8:** Performance characteristics of various models for RA classification.

Models	Radiographs				Thermal Images			
	Cohen’s Kappa	TPR	FPR	Execution Time (s)	Cohen’s Kappa	TPR	FPR	Execution Time (s)
AlexNet	0.41	0.69	0.11	263	0.43	0.71	0.13	270
Xception	0.40	0.72	0.13	270	0.75	0.92	0.29	327
ResNet152V2	0.75	0.89	0.14	574	0.80	0.90	0.18	540
RA-XTNet	0.80	0.87	0.25	255	0.85	0.94	0.09	248
ViT	0.60	0.80	0.20	1603	0.80	0.14	0.95	1646

**Table 9:** Comparison of the existing literature with the proposed model for the prediction of RA.

Author	Input to the Model	Model	Accuracy
Bai et al. [11]	Clinical values	ANN	90.6%
Morita et al. [12]	Hand radiographs	Custom CNN	74.1%
Ureten et al. [13]	Hand radiographs	Custom CNN	73.33%
Ho et al. [19]	Hand thermal images	Adaboost	87.5%
Morales-Ivorra et al. [20]	Hand thermal images	k-NN	79%
Kumar et al. [22]	Hand thermal images	CNN-LSTM	92.78%
Bonnin et al. [41]	Hand X-rays	Custom CNN	84%
Folle et al. [42]	CT images	Convolutional supervised autoencoder	86%
Stolpovsky [43]	Hand X-rays	RheumaViT	67% for joint and 74% for erosion
Proposed work	RA-XTNet	Hand radiographs Hand thermal images	90% 92.5%

## Data Availability

The data presented in this study are available on request from the corresponding author.
